# Stemona alkaloid derivative induce ferroptosis of colorectal cancer cell by mediating carnitine palmitoyltransferase 1

**DOI:** 10.3389/fchem.2024.1478674

**Published:** 2024-10-03

**Authors:** He Yang, Ling Wang, Mengcheng Zhang, Xingkang Wu, Zhenyu Li, Kaiqing Ma

**Affiliations:** ^1^ Key Laboratory of Chemical Biology and Molecular Engineering of Ministry of Education, Institute of Molecular Science, Shanxi University, Taiyuan, China; ^2^ Modern Research Center for Traditional Chinese Medicine, Shanxi University, Taiyuan, China

**Keywords:** acylcarnitine, ferroptosis, CPT-1, colorectal cancer, stemona alkaloid

## Abstract

Accumulation of acylcarnitines is a characteristic feature of various metabolic disorders affecting fatty acid metabolism. Despite extensive research, no specific molecules have been identified to induce ferroptosis through the regulation of acylcarnitine metabolism. In this study, acylcarnitine accumulation was identified based on cell metabolomics study after the treatment with Stemona alkaloid derivative (SA-11), which was proved to induce ferroptosis in our previous research. Furthermore, the CPT-1 level was proved to significantly increase, while the CPT-2 level indicated no significant difference, which resulted in the accumulation of acylcarnitine. Besides, the ferroptosis-inducing ability of SA-11 was significantly enhanced by the addition of exogenous acylcarnitine, presumably due to the production of additional ROS. This hypothesis was corroborated by the observation of increased ROS levels in HCT-116 cells treated with SA-11 compared to the control group. These findings suggest that targeting acylcarnitine metabolism, particularly through CPT-1, may offer a novel therapeutic strategy for cancer treatment by enhancing ferroptosis induction.

## 1 Introduction

Natural products and their derivatives have been an invaluable source for drug discovery (Newman and Cragg, 2020; Atanasov et al., 2021). Furthermore, natural products serve as valuable tools for the discovery of drug targets and the elucidation of disease mechanisms (Zhou and Xiao, 2018). However, identifying functional targets and clarifying the molecular mechanism of action of bioactive natural products and their derivatives have been proven to be particularly challenging (Laraia et al., 2018; Chen et al., 2020). Metabolomics, a powerful analytical approach that profiles and quantifies small-molecule metabolites within biological systems, offers unique insights into the biochemical pathways and processes affected by the bioactive molecules (Pang and Hu, 2023; Zhao et al., 2018; Beyoğlu and Idle, 2020). Notably, berberine was identified to inhibit pancreatic cancer cell viability and metastasis by regulating citrate metabolism based on cell metabolomics (Liu et al., 2020). Meanwhile, ponicidin was found to suppress pancreatic cancer growth through ferroptosis based on the metabolomics research (Cui et al., 2022). Recently, scutellarin was demonstrated to regulate the upstream metabolic pathway of mitochondrial oxidative phosphorylation (OXPHOS)and electron transport chain through metabolomics studies (Sheng et al., 2023).

**FIGURE 1:**
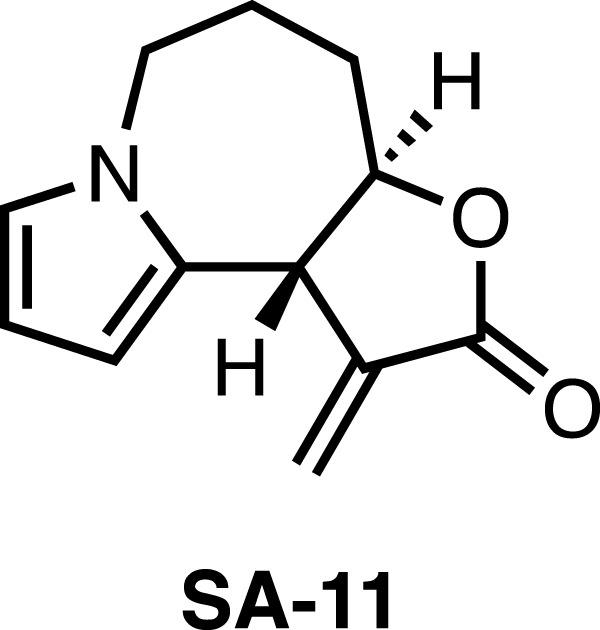
The structure of SA-11.

Recently, we took note of Stemona alkaloids, which possess diverse structures and interesting biological activities such as anticancer (Greger, 2019). In our previous study, we completed total syntheses of stemona alkaloids including bisdehydroneostemoninine, bisdehydrostemoninine, and parvistemonine A (Ma et al., 2018; Ma et al., 2019). Subsequently, the derivatives of stemona alkaloids were designed and synthesized, followed by the biological evaluation. The stemona alkaloids derivative SA-11 (Figure 1) was identified to efficiently inhibit various colorectal cancer (CRC) cells at low micromolar, including 5-fluorouracil-resistant CRC cells (Ma et al., 2021). Moreover, SA-11 was demonstrated to induce oxidative stress and inhibit cancer cell growth through ferroptosis (Ma et al., 2022). However, metabolic profiling is still lacking, which is vital to a thorough understanding the action mechanism of SA-11.

The transport of fatty acids into the mitochondria via acylcarnitine is crucial for energy production (Melone et al., 2018). Fatty acid oxidation disorders manifest due to abnormality of the key enzymes participating in the fatty acid oxidation (Ribas and Vargas, 2022). Consequently, the incomplete oxidation of fatty acids occurs, resulting in the accumulation of acylcarnitines within the cell (Dambrova et al., 2022; Knottnerus et al., 2018). Notably, the accumulation of acylcarnitines promotes the production of re-active oxygen species (ROS) in the myocardial cell which causes DNA damage and dysregulated signaling pathways. ROS have been recognized as potent initiators of the peroxidation process in polyunsaturated fatty acids (PUFAs) (Kankuri et al., 2023). Moreover, the excessive formation of acylcarnitines consume the monounsaturated fatty acids, which have been demonstrated to be potent inhibitors of ferroptosis (Magtanong et al., 2019). However, so far, there is no small molecules reported to induce ferroptosis through mediating acylcarnitines metabolism.

In this work, a metabolomics study (Guo et al., 2023; Liu et al., 2022) based on an ultra-high performance liquid chromatography combined with electrospray ionization quadrupole/orbitrap high-resolution mass spectrometry (UPLC-Q-Orbitrap HRMS) was established to investigate the metabolic profiling of HCT-116 cells induced by SA-11 to explore its probable antitumor mechanism. Distinct biomarkers caused by SA-11 were identified and their metabolic pathways were also discussed. Notably, four acylcarnitines including lauroylcarnitine (C12), myristoleoylcarnitine (C14:1), myristoylcarnitine (C14), and palmitoleoylcarnitine (C16:1) displayed significant increases after SA-11 treatment across all time points. Furthermore, the carnitine palmitoyltransferase 1 (CPT-1) level was significantly higher than that in the control group after the cell was treated with SA-11. The SA-11 cytotoxicity was reduced when the CPT-1 inhibitor (etomoxir) was added. Finally, the cytotoxicity of SA-11 was further improved when additional lauroylcarnitine (C12) was added. Herein, we demonstrated that SA-11 induced ferroptosis by mediating the metabolism of fatty acid oxidation.

## 2 Results and discussions

### 2.1 LC-MS-based metabolomics analysis

In the previous research, SA-11 was demonstrated to induce the ferroptosis of cancer cells *in vitro* through the effect on the redox balance. The cytotoxicity of SA-11 was performed against different CRCs as well as a normal cell to study SA-11 toxicity (Ma et al., 2021). Furthermore, in the preliminary *in vivo* study, SA-11 has been demonstrated to inhibit tumor growth in a concentration-dependent manner with minimal toxicity (Supplementary Figures S1–S3). However, the effect of SA-11 on metabolic profiling is still lacking, which is vital to a thorough understanding of the antiproliferation action of SA-11. Thus, we conducted metabolomics studies based on the HCT-116 cell lines. In order to elucidate the shifts in metabolic profiling induced by SA-11 treatment and to identify a more comprehensive set of differential metabolites, the incubation time was tested. The results indicated that cell viability was prone to reduce after 8 h, which implied that the metabolites changed significantly after treatment with SA-11 (Supplementary Figure S4). Therefore, a focused analysis was conducted utilizing data from samples subjected to an 8-h treatment.

**FIGURE 2:**
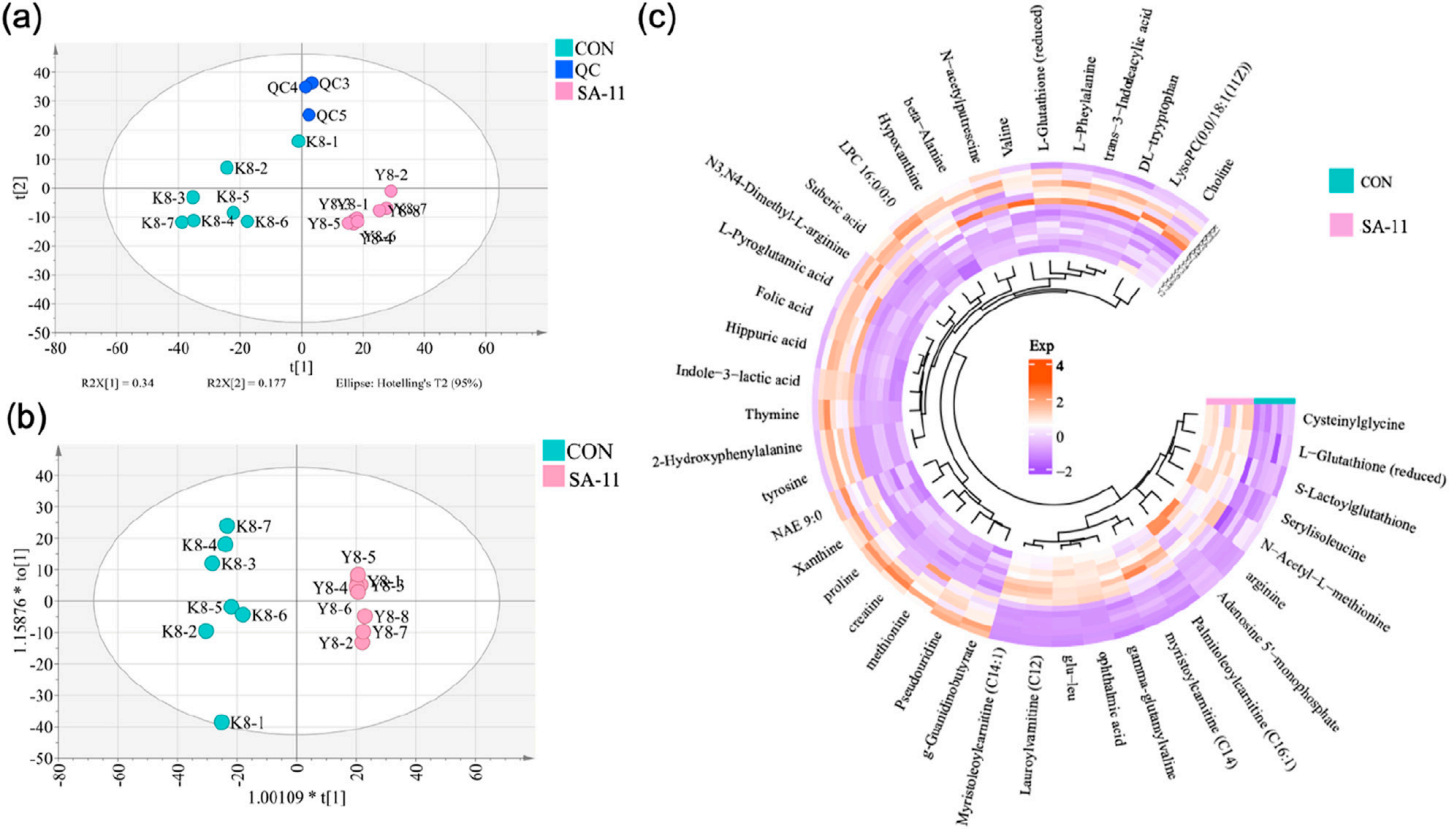
The metabolic profiling analysis. **(A)** PCA score spots of all samples, **(B)** OPLS-DA score spots (CV-ANOVA, *p* = 2.30 × 10^−5^; R2X = 0.9887, Q2 = 0.916), **(C)** The Circos heatmap of the 41 differential metabolites.

A total of 1,577 metabolic features were detected in the positive and negative ion modes, and the relative standard deviation (RSD%) values of all peaks in quality control (QC) samples were lower than 30% (Supplementary Table S1). The data distinctly indicated the stability and reliability of the analytical platform employed. Moreover, the principal components analysis (PCA) score plots effectively demonstrated a distinct separation between the control (CON) and SA-11 treated groups, as evident in Figure 2A. This distinct separation underlined the notable metabolic alterations induced by SA-11 treatment, further emphasizing its potential influence on the cellular metabolome. Then the orthogonal partial least squares discriminant analysis model (OPLS-DA) was further established to screen the differential metabolites (Figure 2B), and based on the threshold of VIP >1, *p* < 0.05, and FC > 1.2 or <0.8, a total of 41 endogenous metabolites were filtered out as the key metabolites contributing to the anti-cancer effect of SA-11 (Supplementary Table S2), which mainly including fourteen amino acids, eight peptides, four acylcarnitines, two indoles, two nucleosides, two pholipids and two purines.

The nuanced interplay and alterations within the cohort of 41 differential metabolites were vividly portrayed utilizing a Circos heatmap, as depicted in Figure 2C. Following SA-11 treatment, a discernible upregulation was observed in 14 metabolites, mainly including four acylcarnitines: lauroylcarnitine (C12), myristoleoylcarnitine (C14:1), myristoylcarnitine (C14), and palmitoleoylcarnitine (C16:1). Conversely, a substantial decrease was evident in 27 metabolites, including thymine, L-glutathione oxidized, xanthine, and creatine. The identified key metabolites shed light on the specific bio-chemical shifts implicated in the anti-cancer effects of SA-11.

### 2.2 Correlation network analysis of the differential metabolites

**FIGURE 3:**
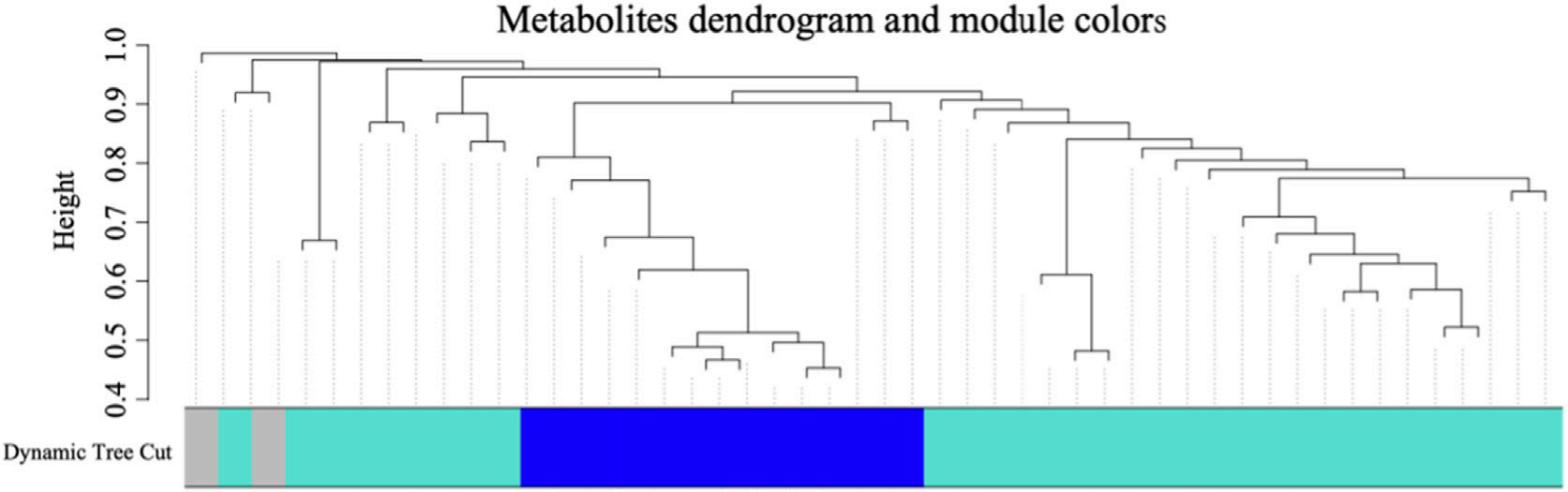
The cluster dendrogram of the 41 differential metabolites and metabolite clusters in different colors represented different co-expression modules.

**FIGURE 4:**
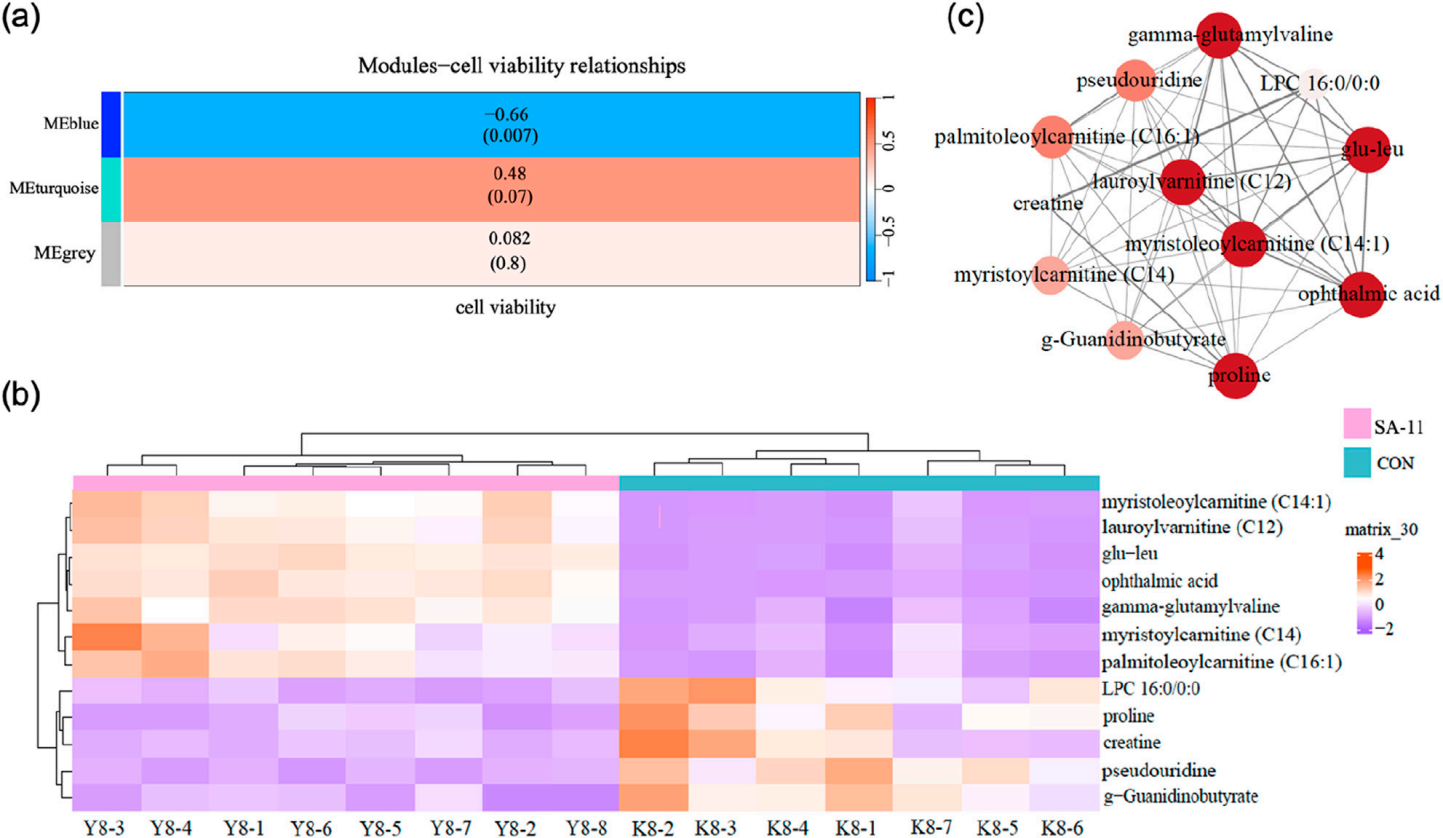
Network construction of consensus modules based on WGCNA. **(A)** Spearman’s correlation analysis between the modules and cell viability, the table was color-coded by correlation according to the color legend, and the correlation coefficients and *p*-value were displayed in each cell. **(B)** The heatmap of metabolites in the blue module. **(C)** Networks of metabolites in the blue model and the hub metabolites were indicated by larger circles and darker colors.

In order to discern metabolites exhibiting highly synergistic changes, a weighted correlation network analysis (WGCNA) was conducted based on the cohort of 41 differential metabolites, which identifying three distinct metabolite modules (Figure 3). Then we sought to explore the hub module’s contribution to the anti-cancer effect of SA-11, focusing on the co-expression modules and their correlation with cell viability using Spearman’s correlation analysis (Figure 4A). Specifically, we set criteria of *p* < 0.05 and a correlation coefficient (r) of ≥0.5 or ≤ −0.5 to establish significant correlations. Notably, only the blue module exhibited a significant negative correlation with cell viability (*p* = 0.007, r = −0.66). Meanwhile, the blue module encompassed 12 metabolites, featuring a spectrum of four acylcarnitines, four peptides, two amino acids, one nucleoside, and one phospholipid. We analyzed the metabolites within this blue module and constructed heatmaps, effectively visualizing the metabolic differences between the CON and SA-11 treated groups (Figure 4B). Myristoleoylcarnitine (C14:1), lauroylcarnitine (C12), palmitoleoylcarnitine (C16:1), myristoylcarnitine (C14), and ophthalmic acid showed significant increases after SA-11 treatment. Furthermore, we conducted Spearman correlation analysis among metabolites within the blue module to identify hub metabolites (Figure 4C). Larger and darker nodes indicated higher node degrees, while thicker lines represented stronger correlation weights. Interestingly, myristoleoylcarnitine (C14:1), lauroylcarnitine (C12), and ophthalmic acid exhibited significant correlations with other metabolites in the network. Ophthalmic acid was proven to be an oxidative stress biomarker in the previous research (Soga et al., 2006; Skouta et al., 2014). Indeed, in our previous work, SA-11 was found to consume the thiol in the mitochondria and induce oxidative stress, which eventually caused ferroptosis.

**FIGURE 5:**
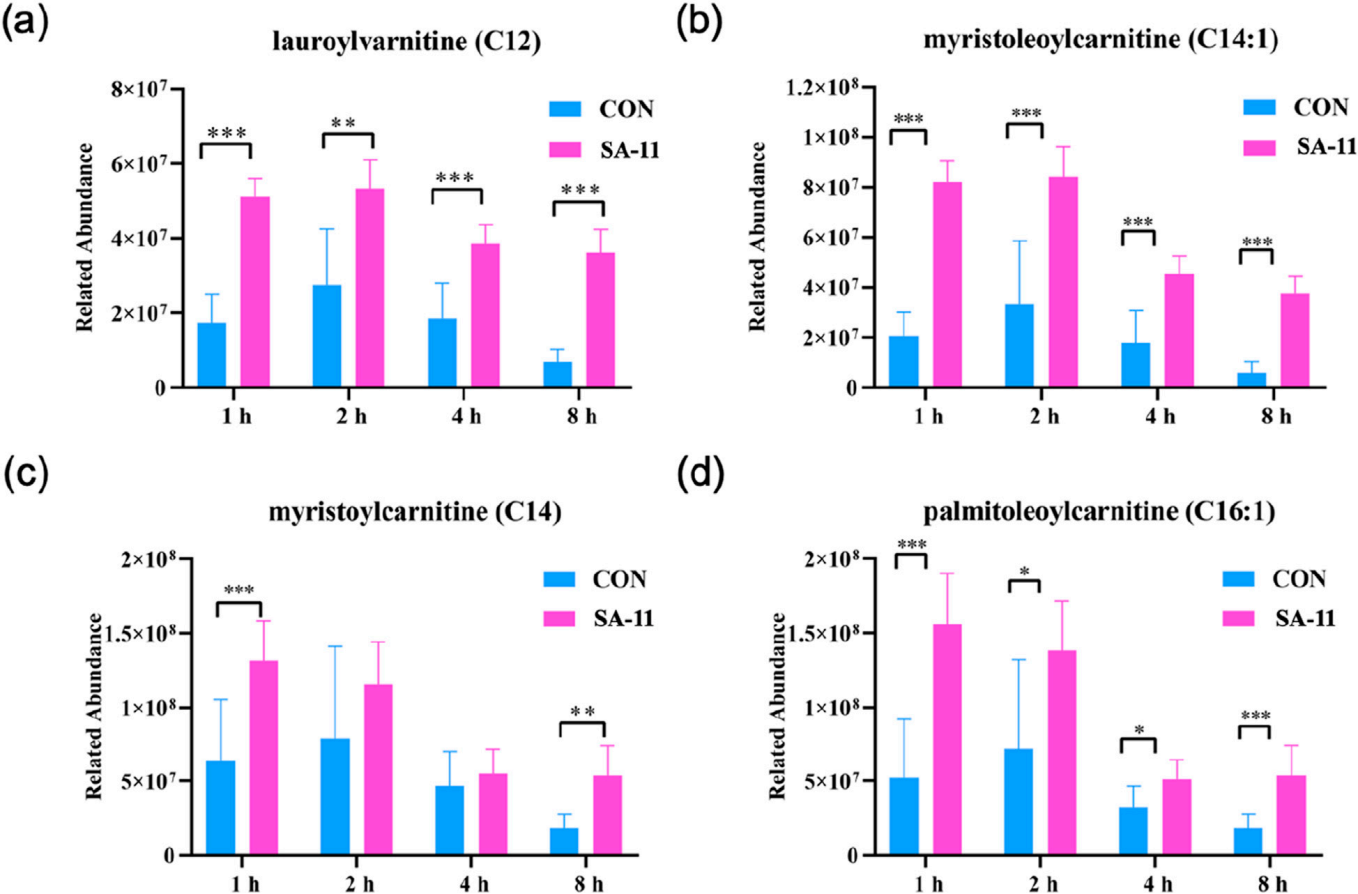
The level of acylcarnitine treated with or without SA-11. **(A)** lauroylcarnitine (C12), **(B)** myristoleoylcarnitine (C14:1), **(C)** myristoylcarnitine (C14) and **(D)** palmitoleoylcarnitine (C16:1) in the control and SA-11 groups. Longitudinal coordinates represent the relative content of genera. All data are expressed as mean ± SD (n = 5 * *p* < 0.05, ** *p* < 0.01, *** *p* < 0.001).

Acylcarnitines, derivatives of fatty acids, are involved in fatty acid metabolism and mitochondrial β-oxidation. Dysregulation of fatty acid metabolism can lead to the accumulation of acylcarnitines, potentially impacting cellular redox balance and contributing to the pathogenesis of various diseases, including those associated with ferroptosis. Thus, we focused on the changes in the four acylcarnitines after varying treatment durations (1, 2, 4, and 8 h) by presenting their relative abundances using bar charts (Figure 5). Strikingly, lauroylcarnitine (C12), myristoleoylcarnitine (C14:1), myristoylcarnitine (C14), and palmitoleoylcarnitine (C16:1) displayed significant increases after SA-11 treatment across all time points. This noteworthy observation underscores the putative significance of acylcarnitines in modulating redox balance in response to SA-11 exposure.

### 2.3 The mechanism verification

**FIGURE 6:**
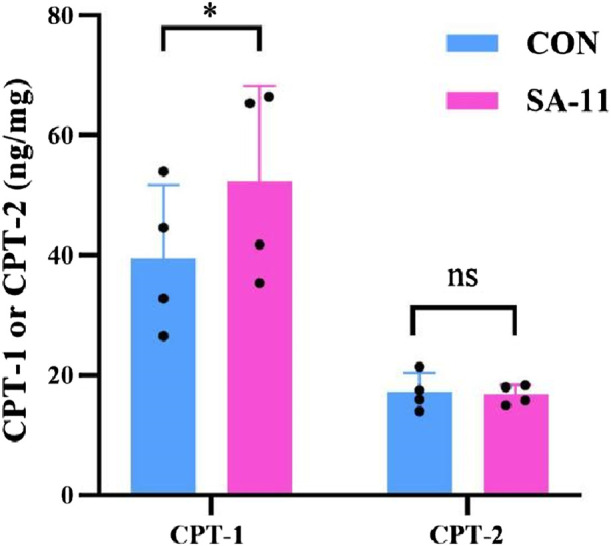
Protein level of CPT-1 or CPT-2 after 4 h of treatment HCT-116 cells with 10 μM SA-11 (n = 4 independent biological replicates). All data are presented as mean ± SD. *p < 0.05; ns, no significant.

CPT-1 and CPT-2 are involved in lipid metabolism, specifically in the transport of fatty acids into mitochondria for energy production. CPT-1 converts fatty acyl-CoA into acylcarnitine, allowing it to be transported across the mitochondrial membranes. Meanwhile, CPT-2 reverses the action of CPT-1, converting acylcarnitine back to acyl-CoA, which enters the β-oxidation pathway. To investigate the observed substantial increase in acylcarnitines in our metabolomics data, we examined the protein expression levels of CPT-1 and CPT-2 using enzyme-linked immunosorbent assay (ELISA) kits (Figure 6). Our findings revealed that the protein level of CPT-1 in the SA-11 treated group was significantly higher than that of the control group (*p* < 0.05). This result was further verified by the Western blot assay (Supplementary Figure S5). In contrast, there was no significant difference in the protein level of CPT-2 between the SA-11 treated group and the control group. This suggests that treatment with SA-11 leads to an increase in acylcarnitines, primarily through the upregulation of CPT-1, without impacting the expression of CPT-2.

**FIGURE 7:**
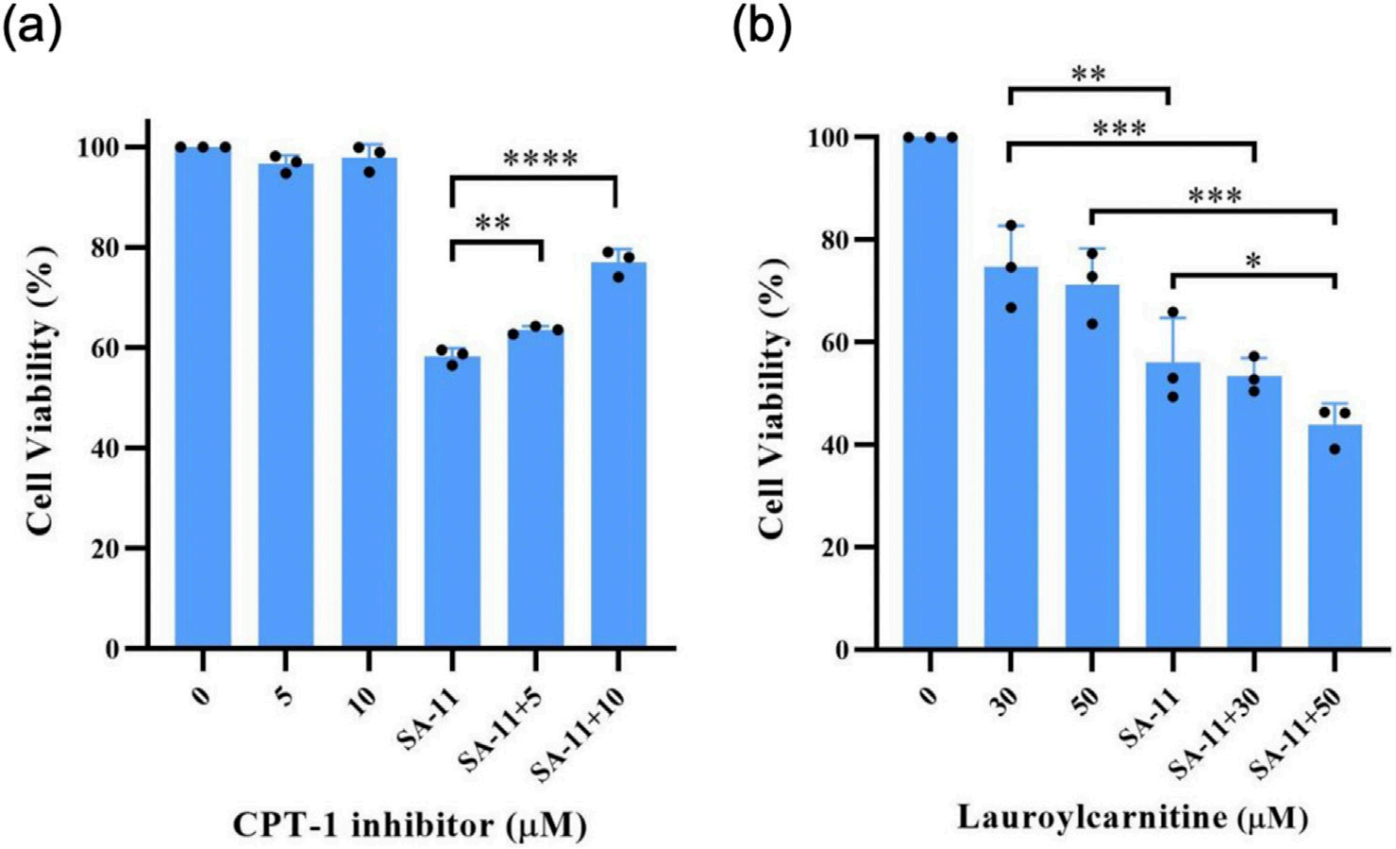
The effect of CPT-1 inhibitor and lauroylcarnitine on the HCT-116 cells in 24 h **(A)** Effect of CPT-1 inhibitor on cell viability induced by SA-11 (20 μM). Data are represented as mean ± SD. **(B)** Effect of lauroylcarnitine on cell viability followed by treatment with SA-11 (20 μM). Data are represented as mean ± SD (n = 3).

To substantiate the pivotal involvement of CPT-1 in SA-11-induced ferroptosis, we conducted cell viability assays utilizing etomoxir, a well-established inhibitor of CPT-1 (Figure 7). Surprisingly, etomoxir alone demonstrated no discernible effect on the cell viability of HCT-116 cells, whereas SA-11 significantly diminished cell viability. Notably, upon incubation with SA-11, an escalation in cell viability was observed with increasing concentrations of etomoxir. This observation provides further evidence supporting the proposition that the cytotoxic effects of SA-11 may indeed be closely associated with the modulation of CPT-1 activity. Given the observed ability of SA-11 to induce an increase in endogenous acylcarnitines and subsequently induce ferroptosis, we postulated that the addition of exogenous acylcarnitines might potentiate the cytotoxic effects of SA-11. Our investigation unveiled that lauroylcarnitine (C12) alone exhibited a weak degree of cytotoxicity. However, upon co-administration with SA-11, lauroylcarnitine (C12) demonstrated a concentration-dependent reduction in cell viability. These findings suggest a potential role for acylcarnitines in modulating the cytotoxicity elicited by SA-11.

**FIGURE 8:**
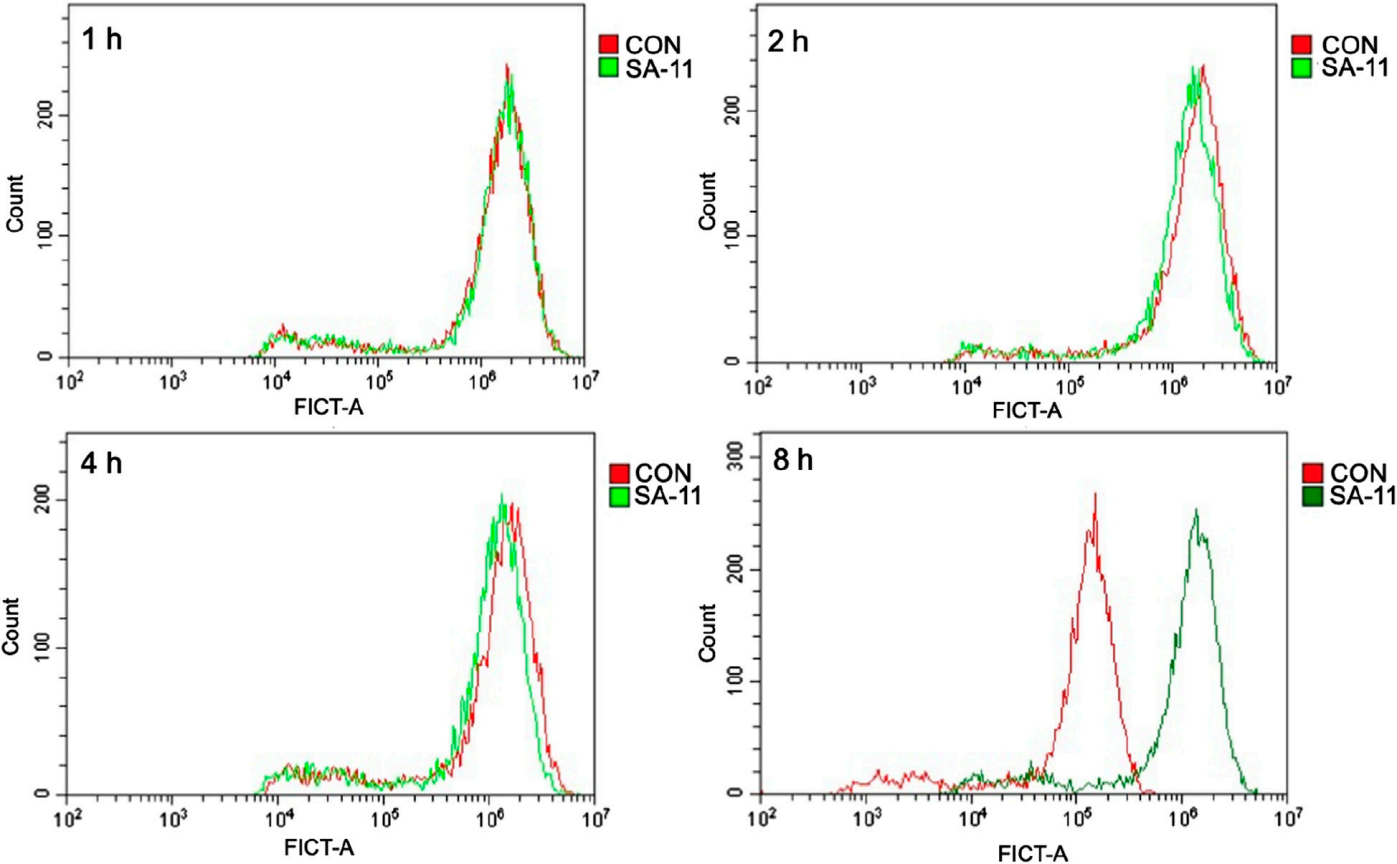
SA-11 elevated ROS in CRC cells. HCT-116 cells were placed in a six-well plate and cultured in a 5% CO_2_ incubator at 37°C for 24 h, and treated with SA-11 for 1, 2, 4, and 8 h, respectively. Fluorescence intensity was measured by flow cytometry.

To investigate the impact of SA-11 on intracellular ROS levels, a series of experiments was conducted on the HCT-116 colon cancer cell line. Following exposure to varying durations of SA-11 treatment (1, 2, 4, and 8 h), HCT-116 cells were subjected to incubation with the 2′, 7′-dichlorofluorescein diacetate fluorescent probe, a widely employed ROS indicator. Subsequently, the ROS levels were quantified using flow cytometry. The results, as depicted in Figure 8, revealed a conspicuous augmentation in ROS levels within HCT-116 cells subsequent to an 8-h treatment with SA-11. These findings were consistent with the observations made in cell survival assessments (Supplementary Figure S4). This discernment suggests that SA-11 has the capacity to elevate ROS levels within colon cancer cells. Consequently, it proposes a plausible mechanistic pathway whereby SA-11 mediates the inhibition of colon cancer cell proliferation through the modulation of intracellular acylcarnitine levels, consequently inducing ROS accumulation.

### 2.4 Discussion

The present study showed that the SA-11, a novel stemona alkaloids derivative, could efficiently inhibit CRC cells at low micromolar. Further cell metabolomics research indicated metabolic alteration of the CRC cells responded to SA-11 treatment through fatty acid oxidation, especially lauroylcarnitine (C12), myristoleoylcarnitine (C14:1), myristoylcarnitine (C14), and palmitoleoylcarnitine (C16:1) significant increasing after SA-11 treatment across all time points. In our endeavor to elucidate the underlying mechanism driving the accumulation of acylcarnitines, we observed a notable increase in the protein level of CPT-1 in the SA-11 treated group. Conversely, we did not detect any significant difference in the protein levels of the CPT-2. These results indicated that the anti-cancer mechanism of SA-11 might related to the fatty acid oxidation pathway.

In prior investigations, the anti-tumor efficacy of SA-11 was established, notably through its induction of oxidative stress, thereby impeding cancer cell growth via ferroptosis. Meanwhile, the accumulation of acylcarnitines was observed in the metabolomics study. The accumulation of acylcarnitines has been reported to trigger heightened ROS production in myocardial cells, resulting in DNA damage (Qu et al., 2016; Tan et al., 2021). ROS, acknowledged as potent initiators of peroxidation processes in PUFAs, play a crucial role in ferroptosis induction (Zhang et al., 2023). Furthermore, the excessive formation of acylcarnitines consume the monounsaturated fatty acids, which have been demonstrated to be potent inhibitors of ferroptosis (Wang et al., 2020; Jiang et al., 2021).

In efforts to further delineate the role of CPT-1 in SA-11’s anti-cancer mechanism, we observed a significant reduction in SA-11-induced cytotoxicity against HCT-116 cells upon co-administration with a CPT-1 inhibitor. Additionally, exogenous acylcarnitines were found to enhance the cytotoxic effects of SA-11. Further substantiating SA-11’s mechanism, treatment of HCT-116 cells with SA-11 for 8 h revealed a notable increase in ROS production, as detected by the DCFH-DA fluorescent probe.

However, while our current investigations have established a correlation between SA-11’s anti-cancer effect in colorectal cancer cells and fatty acid oxidation, the precise mechanism by which fatty acid oxidation precipitates ferroptosis in tumor cells remains elusive. Ongoing research endeavors in our laboratory aim to elucidate the mechanisms underlying the upregulation of CPT-1 induced by SA-11, with forthcoming findings anticipated to provide additional insights into SA-11’s anti-cancer properties.

## 3 Materials and methods

### 3.1 Cell viability assay

Cell viability was assessed by sulforhodamine B assays as described. Cells were seeded in 96-well plates and treated with different concentrations of the compound for a certain time. Cells were fixed with 10% trichloroacetic acid at 4°C. The plate was rinsed five times with slow-running water and dried at room temperature. After drying, the cells were stained for 25 min with a 0.4% SRB solution, and the unbound dye was rinsed five times with 1% acetic acid. The protein-bound dye was dissolved in a 10 mM Tris solution (pH 10.5), and the OD value was measured at 510 nm in a microplate reader cell inhibition (%) was calculated.

### 3.2 Cell reactive oxygen species detection

HCT-116 cells were placed in a six-well plate and cultured in a 5% CO_2_ incubator at 37°C for 24 h, and treated with SA-11 for 1, 2, 4, and 8 h, respectively. Subsequently, the cells were rinsed with PBS, and after digestion and centrifugation, the cell suspension was obtained. A 5 μM reactive oxygen species fluorescent probe (DCFH-DA) was added and incubated at room temperature for 20 min. After centrifugation, washed twice with PBS, and then PBS containing 1% fetal bovine serum was added for suspension. Fluorescence intensity was measured by flow cytometry.

### 3.3 Metabolomics analysis

Following specified incubation durations of 1, 2, 4, and 8 h with SA-11, metabolites were extracted from cellular samples using a previously established protocol with minor adjustment (Cao et al., 2021), then a Thermo Fisher U3000 UPLC system was used for sample analysis (Wang et al., 2019), the details of the metabolites extract and UPLC-Q-Orbitrap HRMS parameters were described in Supplementary Material Part 1. For metabolite identification, we employed a combined approach using Xcalibur software (version 3.2) and MS-DIAL (version 4.90), as described in the previous study (Wang et al., 2024). The correlation network analysis of the differential metabolites was established employing the R package “WGCNA” (version: WGCNA_1.70–3) and the “Cytoscape” package, and the details were shown in Supplementary Information Part 2.

### 3.4 Statistical analysis

A t-test was conducted using GraphPad Prism, a widely recognized statistical analysis software. The resultant data are presented in the standard format of mean ± standard deviation (SD). The predetermined significance level for this analysis was set at *p* < 0.05.

## 4 Conclusion

The accumulation of acylcarnitines, potentially stemming from altered fatty acid oxidation, alongside the observed elevation in ROS levels, may constitute pivotal mechanisms underlying SA-11’s anticancer properties. Our study offers new insights into potential therapeutic strategies for colorectal cancer treatment, suggesting that targeting CPT-1 in cancer cell could be beneficial. The evidence also indicates that SA-11 holds considerable promise for colorectal cancer therapy with novel mechanism.

## Data Availability

The original contributions presented in the study are included in the article/Supplementary Material, further inquiries can be directed to the corresponding authors.
