# Supplementary material for: Stemona alkaloid derivative induce ferroptosis of colorectal cancer cell by mediating carnitine palmitoyltransferase 1
Source: Front Chem. 2024 Oct 3;12:1478674. doi: 10.3389/fchem.2024.1478674 (PMC11484037; doi:10.3389/fchem.2024.1478674)
Supplement: Supplementary file 2 [file DataSheet1.docx]

Stemona alkaloid derivative induce ferroptosis of colorectal cancer cell by mediating carnitine palmitoyltransferase 1

He Yang ^1, a^, Ling Wang ^2, a^, Mengcheng Zhang ^2^, Xingkang Wu^2^, Zhenyu Li ^2,*^ and Kaiqing Ma^1,*^

^1^Key Laboratory of Chemical Biology and Molecular Engineering of Ministry of Education, Institute of Molecular Science, Shanxi University, Taiyuan 030006, China, ^2^Modern Research Center for Traditional Chinese Medicine, Shanxi University, Taiyuan 030006, China, ^a^ These authors contributed equally to this work

^*^Correspondence authors: Zhenyu Li lizhenyu@sxu.edu.cn; Kaiqing Ma, [makaiqing@sxu.edu.cn](mailto:makaiqing@sxu.edu.cn)

**Part 1. Sample preparation and UHPLC-TOF/MS parameters.**

**Sample Preparation**

Following specified incubation durations of 1, 2, 4, and 8 hours with **SA-11**, metabolites were extracted from cellular samples using a previously established protocol with minor adjustments^[1]^.

To elucidate briefly, the culture medium was carefully aspirated, and cells were subsequently rinsed with cold phosphate-buffered saline (PBS). Subsequently, 1 mL of ice-cold 80% methanol was added to each sample. Post-incubation at -80 °C overnight, the cells were meticulously scraped and transferred into 1.5 mL tubes. The cellular suspensions underwent ultrasonication for 5 minutes within an ice water bath, followed by centrifugation at 13,000 rpm at 4 °C for 15 minutes. The resulting supernatant was subjected to freeze-drying under vacuum conditions. The ensuing residue was reconstituted in 80 μL of 80% methanol containing 0.1% formic acid. Vigorous vortex-mixing for 30 seconds ensued, succeeded by ultrasonic extraction for 10 minutes in an ice water bath. Subsequently, the samples were centrifuged at 13,000 rpm at 4 °C for 10 minutes to obtain the supernatant designated for metabolomics analysis. Quality control (QC) samples, pivotal for methodological calibration, were meticulously prepared by amalgamating equal volumes of each individual sample.

**Instruments and liquid chromatography-tandem mass spectrometry (UPLC/MS–MS) analysis**

The analytical component of this study entailed the utilization of a Thermo Fisher U3000 UPLC system for meticulous sample analysis. The chromatographic separation of analytes was accomplished through a Waters Acquity UHPLC HSS T3 column (2.1 mm×100 mm, 1.8 μm). The following shows the details of UPLC-Q-Orbitrap HRMS parameters ^[2]^. The mobile phase, an instrumental factor of utmost importance, comprised two constituents: (A) a 0.1% formic acid aqueous solution, and (B) acetonitrile. The elution process was executed using a gradient approach meticulously optimized according to established protocols. Specifically, the gradient elution transpired as follows: 0 to 2 minutes with 1 % to 1% B; 2 to 8 minutes with 1% to 35% B; 8 to 11 minutes with 35% to 40% B; 11 to 14 minutes with 40% to 60% B; 14 to 17 minutes with 60% to 99% B; 17 to 18 minutes with 99% B;18 to 19 minutes with 99% to 1% B; and 19 to 22 minutes, maintaining with 1% B. Maintaining the column at a consistent temperature of 40 °C was essential for optimal separation. Additionally, the samples were stored in the autosampler at a controlled temperature of 4 °C to preserve their integrity. For each injection, a precise volume of 5 µL was employed to ensure accuracy and reproducibility.

**MS conditions**

The mass spectrometric analysis was conducted using a state-of-the-art Thermo Scientific ^TM^ Q Exactive hybrid quadrupole-orbitrap mass spectrometer (Thermo Fisher Scientific, USA). The acquired mass data were diligently recorded for each sample in both positive and negative ionization modes, encompassing a comprehensive spectral range. Key instrumental parameters were meticulously configured to ensure optimal data acquisition and resolution. For the full MS scan mode, the following parameters were employed: a resolution of 70,000, capturing a scan range spanning from 70 to 1050 m/z. The subsequent data-dependent MS2 (dd-MS^2^) and dd-selected ion monitoring (dd-SIM) modes featured a resolution of 17,500 and an isolation window of 1.4 m/z. Moreover, the normalized collision energy (NEC) settings were precisely tuned to 20, 40, and 60, respectively, facilitating efficient fragmentation of ions for in-depth structural analysis. The optimized HESI parameters were as follows: a probe heater temperature set to 300 °C, a capillary temperature maintained at 320 °C, and spray voltages of 3.5 kV (positive mode) and 2.5 kV (negative mode) were applied. Additionally, the sheath gas and auxiliary gas flow rates were finely tuned to 35 psi and 10 psi, respectively, in alignment with best practices in the field.

**Data Processing and Metabolite Identification**

The acquired raw files were processed using Compound Discover 3.2 (CD) to generate datasets of metabolic features. Subsequently, comprehensive analyses were conducted to discern patterns and variations within the data. Principal Component Analysis (PCA) and Orthogonal Projection to Latent Structure Discriminant Analysis (OPLS-DA) were carried out employing SIMCA software (version 14.1). Differential metabolites were filtered based on predefined criteria: a Variable Importance in Projection (VIP) score exceeding 1, statistical significance at P < 0.05, and a fold change (FC) greater than 1.2 or less than 0.8. For metabolite identification, we employed a combined approach using Xcalibur software (version 3.2) and MS-DIAL (version 4.90), as described in the previous study (The beneficial effect of dietary Astragali Radix was related to the regulation of gut microbiota and its metabolites).

**Part 2. Metabolites Network Visualization.**

A Weighted Gene Co-expression Network Analysis (WGCNA) of the pivotal metabolites was conducted utilizing the R package "WGCNA" (version: WGCNA_1.70-3), The co-expression modules were derived employing a step-wise network construction approach with specified parameters: a soft threshold power of 10 to achieve a scale-free R2 of 0.60 and a minimum module size set to 10. Default values were maintained for other parameters. To identify central metabolites within each module, interactions among metabolites were computed using the "Cytoscape" package, employing a threshold of 0.1. Subsequently, the interactions were visually represented using Cytoscape software (version 3.6.1).

**Part 3. CPT-1/CPT-2 assay**.

The Human CPT-1/CPT-2 ELISA Kit was used for these experiments. HCT-116 cells in six-well plates were treated with or without 10 μM of **SA-11** for 4 h. Subsequently, the cells were collected by centrifugation and then lysed by repeated freezing and thawing. After centrifugation, 50 μL of the supernatant of the sample to be tested was added into the well mixed, and incubated at 37 ℃ for 40 min. Then, wash the board, add 350 μL × lotion to each hole, wash 4-6 times, and print dry on the filter paper. After that, 100 μL of biotinized antibody working solution was added into the sample hole, mixed, and placed at 37 ℃ for 30 min. Repeat the washing operation, then pour 100 μL of SABC compound working liquid into the hole, mix, and place at 37 ℃ for 20 min. After washing the plate again, add 100 μL of TMB color-developing solution, mix, and react in a dark place at 37 ℃ for 10-20 min. Finally, add 100 μL termination solution and mix well. The absorbance at 450 nm was measured and used to calculate the CPT-1 amount.

**Part 4.** Animal experiments and drug administration

BALB/C nude mice were provided by the Animal Laboratory of Shanxi Cancer Hospital. Animal studies were reported in compliance with the ARRIVE guidelines. At the time of tumor transplantation, the weight of the mice was 21-26 g. The suspension of HCT-116 cells at the logarithmic growth stage was inoculated into the skin of mice. After inoculation, tumor growth was observed and tumor volume was measured periodically. After nine days of inoculation, the mice were randomly divided into five groups: model control group, positive drug group (5-FU, 25 mg/kg), SA-11 high-dose group (40 mg/kg), compound medium-dose group (20 mg/kg) and compound low-dose group (10 mg/kg). Then, the positive drug and the receptor compound SA-11 were injected separately through the abdominal cavity. The dose was administered ten times, five days a week, two days apart. After the experiment, all data were statistically analyzed using SPASS 16.0.


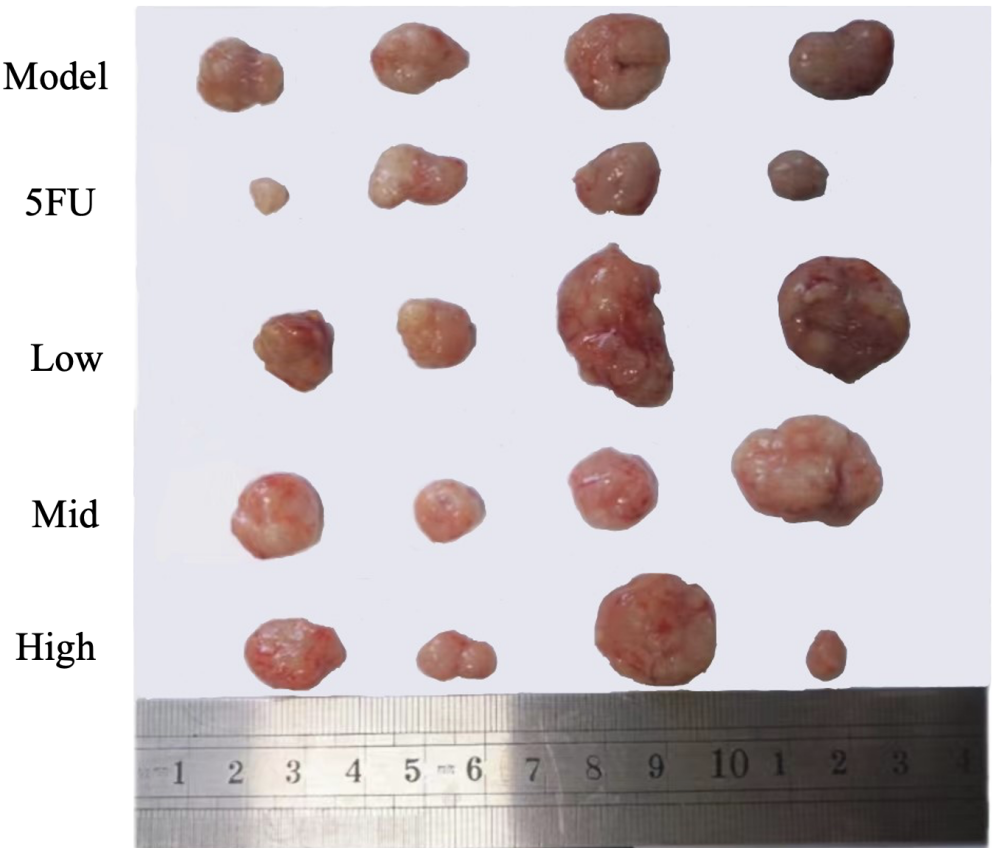


**Fig. S1** The photograph of tumors after five different groups of treatments. Five groups were given intraperitoneal injections of different compounds. Model (injected with PBS only), 5FU (25 mg/kg) only, low dose of SA-11 (10 mg/kg), medium dose of SA-11 (20 mg/kg), high dose of SA-11 (40 mg/kg). The compound SA-11 dissolved in DMSO: EL: saline = 1：1：6.


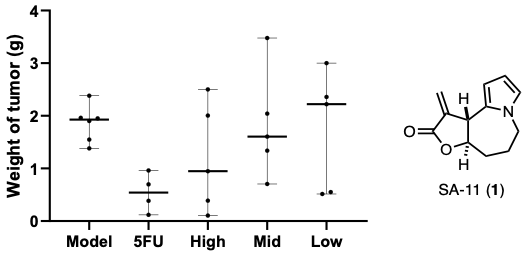


**Fig. S2** **SA-11** Inhibited HCT-116 growth in vivo. Six-to-eight-week-old nude mice were inoculated with 3×106 HCT-116 cells and randomly divided into 5 groups with intraperitoneal injection, the compounds dissolved in DMSO: EL: saline = 1：1：6. Model (injected with PBS only), 5FU (25 mg/kg) only, low dose of **SA-11** (10 mg/kg), medium dose of **SA-11** (20 mg/kg), high dose of SA-11 (40 mg/kg).


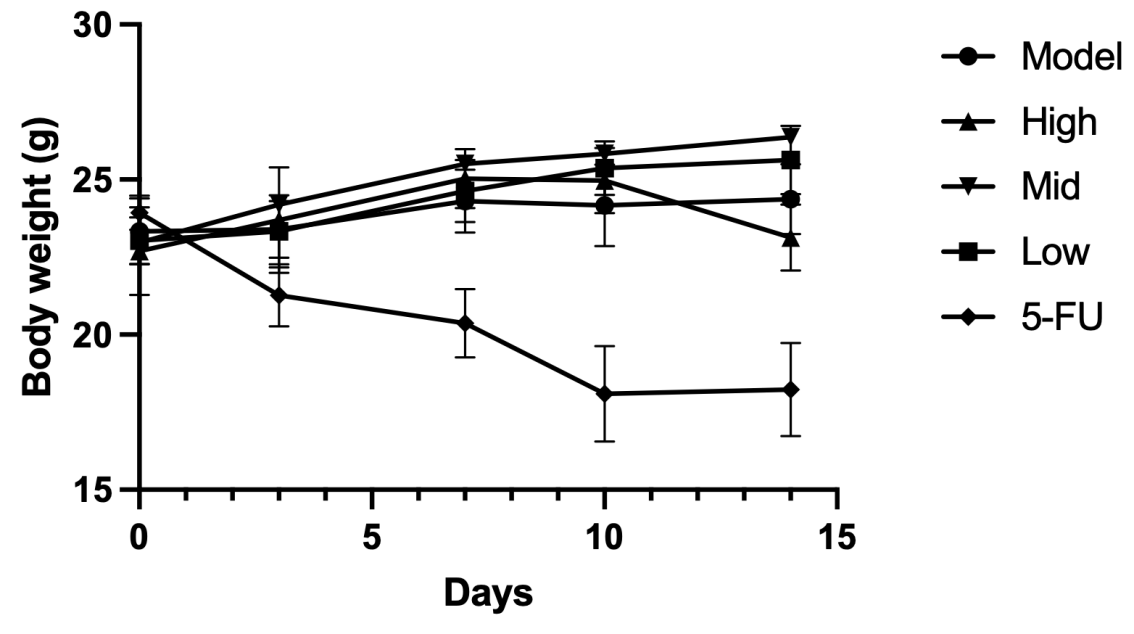


**Fig. S3** Body weight curves of nude mice in each group during treatment with the number of days.


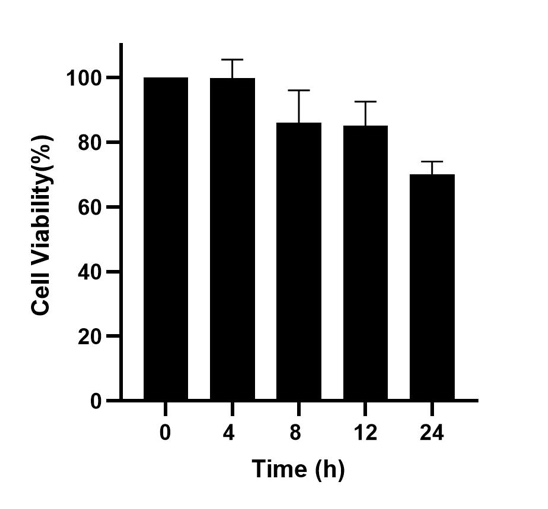


**Fig. S4** The changes in activity of HCT-116 cells incubated with a concentration of 20 µM SA-11 at various time points of 4 hours, 8 hours, 12 hours, and 24 hours were assessed.


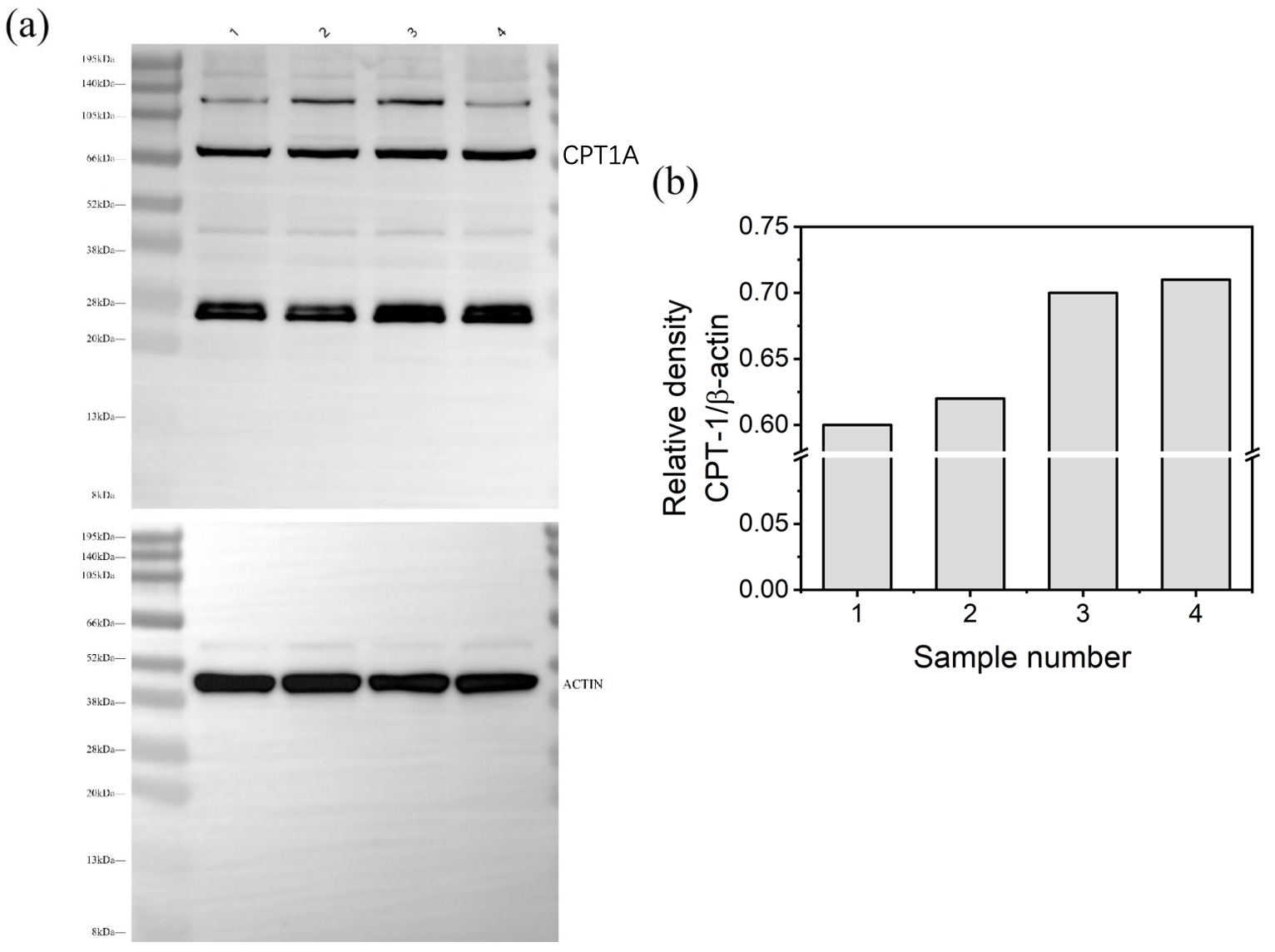


**Fig.S5** Effect of **SA-11** on the expression of CPT-1A protein. (a) WB map of the effect of **SA-11** on CPT-1A protein at 2, 4, and 8 h on HCT-116 cells, respectively. (b) Gray ratio map of CPT-1A protein and internal reference protein actin. (1 represents the control group, 2, 3, and 4 represent the SA-11 incubated cells for 2, 4, 8 h, respectively.)


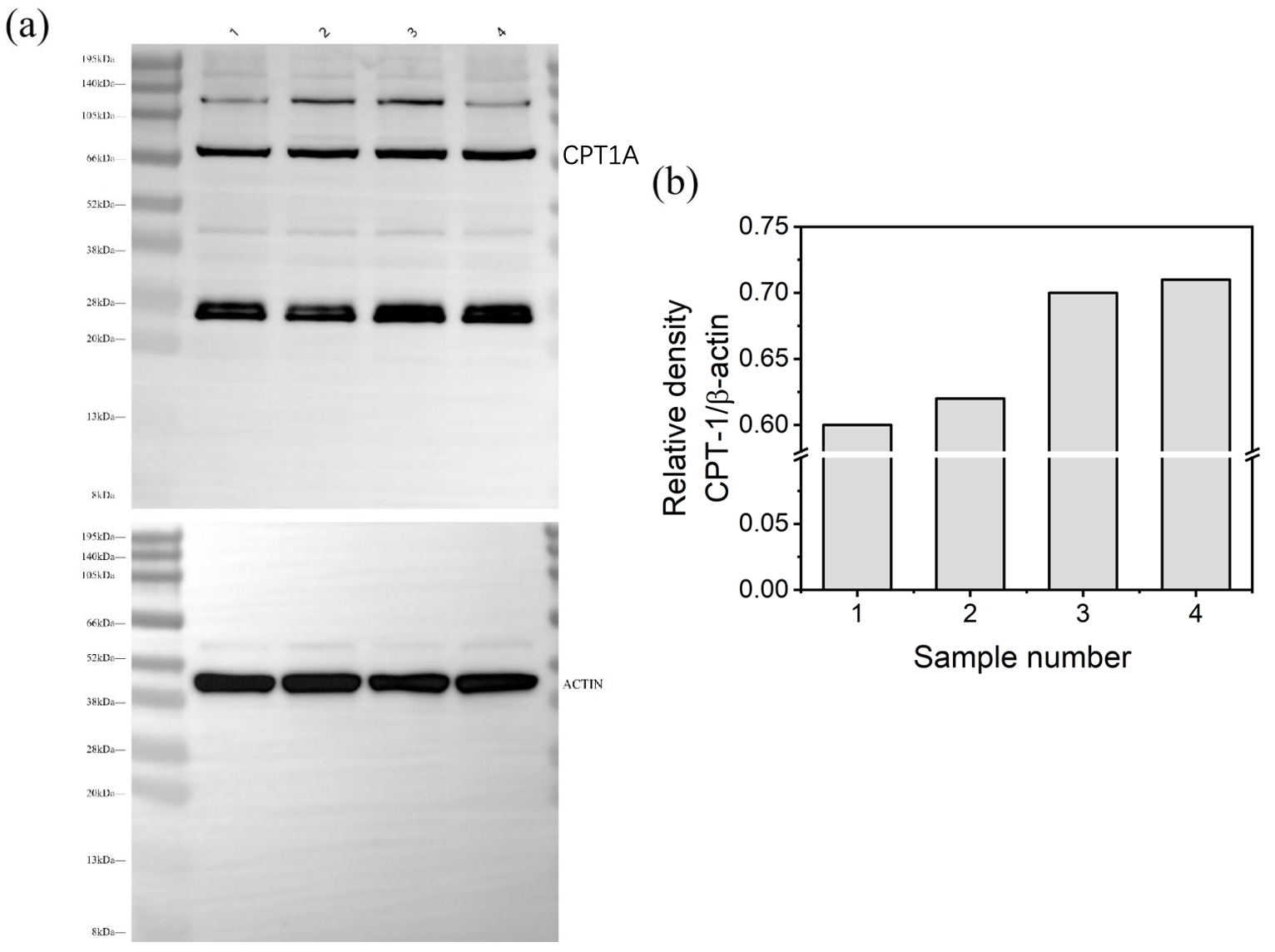


**Fig. S5** Effect of SA-11 on the expression of CPT-1A protein. (a) WB map of the effect of SA-11 on CPT-1A protein at 2, 4, and 8 h on HCT-116 cells, respectively. (b) Gray ratio map of CPT-1A protein and internal reference protein actin. (1 represents the control group, 2, 3, and 4 represent the SA-11 incubated cells for 2, 4, 8 h, respectively.)

**Table S2** Content of 41 differential metabolites between CON and **SA-11** group, and ↑ means the abundance of metabolites was increased, while ↓ means the abundance of metabolites was decreased (* *P* < 0.05, ** *P* < 0.01, *** *P* < 0.001)

| **No** | **compound** | **Molecular formula** | **Ion mode** | **m/z** | **RT** | **MS/MS** | **HMDB** | **FC** | **Type** | **AS-II/CON** |
| --- | --- | --- | --- | --- | --- | --- | --- | --- | --- | --- |
| 1 | Lauroylvarnitine (C12) | C19H37O4N | [M+H]+ | 16.1448 | 344.2791 | 344.2794,144.1017,85.0288,60.0814 | HMDB0002250 | 6.27 | acylcarnitine | ***↑ |
| 2 | Myristoleoylcarnitine (C14:1) | C21H39O4N | [M+H]+ | 16.5358 | 370.2949 | 370.2952,85.0288 | HMDB0240588 | 7.35 | acylcarnitine | ***↑ |
| 3 | Palmitoleoylcarnitine (C16:1) | C23H43O4N | [M+H]+ | 17.3606 | 398.3262 | 398.3251,144.1021,85.0289,60.0814 | HMDB0240782 | 3.21 | acylcarnitine | ***↑ |
| 4 | myristoylcarnitine (C14) | C21H41O4N | [M+H]+ | 17.1447 | 372.3104 | 372.3107,85.0289 | HMDB0254979 | 2.96 | acylcarnitine | ** ↑ |
| 5 | N3,N4-Dimethyl-L-arginine | C8H18N4O2 | [M+H]+ | 1.6061 | 203.1503 | 203.1501,172.1076,158.1289,116.0708,115.0871,88.0874,70.0657 | HMDB0003334 | 0.58 | amino acids | ***↓ |
| 6 | arginine | C6H14N4O2 | [M+H]+ | 1.4156 | 175.1189 | 175.1188,158.0922,116.0708,130.0978,70.0657, | HMDB0000517 | 7.64 | amino acids | ***↑ |
| 7 | L-Pyroglutamic acid | C5H7NO3 | [M+H]+ | 3.0105 | 130.0499 | 130.0500,85.0481,84.0449,56.0501 | HMDB0000267 | 0.59 | amino acids | ***↓ |
| 8 | beta-Alanine | C3H7NO2 | [M+H]+ | 1.5426 | 90.0554 | 90.0554,73.0847,72.0813 | HMDB0000056 | 0.58 | amino acids | * ↓ |
| 9 | L-Phenylalanine | C9H11NO2 | [M-H]- | 6.8536 | 164.0711 | 164.0712,147.0405,119.0494,103.0544,92.0492,72.0081 | HMDB0000159 | 0.62 | amino acids | ** ↓ |
| 10 | methionine | C5H11NO2S | [M+H]+ | 2.4521 | 150.0583 | 150.0577,133.0319,104.0532,102.0554,87.0268,72.0242 | HMDB0028935 | 0.72 | amino acids | ***↓ |
| 11 | proline | C5H9NO2 | [M+H]+ | 1.5426 | 116.0706 | 116.0708,70.0657,72.0813,68.0501 | HMDB0000162 | 0.65 | amino acids | ***↓ |
| 12 | tyrosine | C9H11NO3 | [M+H]+ | 3.6483 | 182.0812 | 182.0806,136.0757,123.0442,119.0493,95.0495,91.0546， | HMDB0000158 | 0.73 | amino acids | ***↓ |
| 13 | Hippuric acid | C9H9NO3 | [M+H]+ | 9.0727 | 180.0654 | 180.064,162.0548,105.0338,106.0373,77.0390,53.0392 | HMDB0000714 | 0.63 | amino acids | ***↓ |
| 14 | Valine | C5H11NO2 | [M+H]+ | 1.4473 | 118.0864 | 118.0865,100.0762,72.0813,70.0657,56.0502, | HMDB0000883 | 0.78 | amino acids | * ↓ |
| 15 | DL-Tryptophan | C11H12N2O2 | [M+H]+ | 7.8745 | 205.0972 | 205.0974,188.0706,159.0916,149.0233,146.0600 | HMDB0030396 | 0.78 | amino acids | * ↓ |
| 16 | 2-Hydroxyphenylalanine | C9 H11 N O3 | [M+H]+ | 3.9875 | 182.0812 | 182.0808,137.0756,119.0492,91.0545 | HMDB0006050 | 0.73 | amino acids | ***↓ |
| 17 | g-Guanidinobutyrate | C5 H11 N3 O2 | [M+H]+ | 2.4555 | 146.0924 | 146.0922,128.0817,87.0448,86.0604,60.0562 | HMDB0003464 | 0.76 | amino acids | ***↓ |
| 18 | N-Acetyl-L-methionine | C7 H13 N O3 S | [M-H]- | 8.3733 | 190.0543 | 190.0539,148.0431,142.0503,112.0395 | HMDB0011745 | 1.22 | amino acids | ** ↑ |
| 19 | choline | C5H13NO | [M+H]+ | 1.4473 | 104.107 | 104.1074,60.0814,59.0736,58.0657 | HMDB0000097 | 0.75 | cholines | * ↓ |
| 20 | Suberic acid | C8H14O4 | [M-H]- | 9.9528 | 173.0814 | 173.0814,155.0710,129.0914,111.0807,59.0129 | HMDB0000893 | 0.42 | fatty acids | ***↓ |
| 21 | Indole-3-lactic acid | C11H11NO3 | [M-H]- | 10.4845 | 204.0663 | 204.0663,186.0555,158.0606,142.0656,130.0654,116.0496,75.0078 | HMDB0000671 | 0.49 | indoles | ***↓ |
| 22 | trans-3-Indoleacrylic acid | C11 H9 N O2 | [M+H]+ | 7.9797 | 188.0706 | 188.0704,142.0650,116.0577,115.0543 | HMDB0000734 | 0.78 | indoles | ** ↓ |
| 23 | NAE 9:0 | C11H23NO2 | [M+H]+ | 3.348 | 202.1802 | 202.1802,62.0607 | / | 0.74 | N-acylethanolamines | * ↓ |
| 24 | Pseudouridine | C9H12N2O6 | [M+H]- | 2.4042 | 243.062 | 243.0621,225.0529,153.0299,111.0190,68.0129 | HMDB0000767 | 0.67 | nucleosides | ***↓ |
| 25 | Adenosine 5'-monophosphate | C10H14N5O7P | [M-H]- | 21.5189 | 346.0558 | 346.0559,192.9911,134.0464,96.9687,78.9581 | HMDB0000045 | 2.73 | nucleosides | * ↑ |
| 26 | ophthalmic acid | C11H19N3O6 | [M+H]+ | 3.0722 | 290.1346 | 290.1340,273.1084,227.1022,161.0921,130.0500,84.0449,58.0658 | HMDB0005765 | 7.63 | peptides | ***↑ |
| 27 | L-Glutathione oxidized | C20H32N6O12S2 | [M+H]+ | 4.1544 | 613.1594 | 613.1588,595.1478,550.1268,538.1277,484.1162,135.0500 | HMDB0003337 | 0.66 | peptides | ** ↓ |
| 28 | L-Glutathione (reduced) | C10H17N3O6S | [M+H]+ | 2.6622 | 308.0911 | 308.0911,291.0651,233.0589,179.0483,130.0499,76.0220,84.0448, | HMDB0000125 | 1.73 | peptides | ***↑ |
| 29 | S-Lactoylglutathione | C13H21N3O8S | [M-H]- | 6.9926 | 378.0978 | 378.0973,360.0765,288.0671,272.0892,177.0333,160.0068,145.0601 | HMDB0001066 | 1.62 | peptides | ***↑ |
| 30 | Serylisoleucine | C9H18N2O4 | [M+H]+ | 7.0455 | 219.134 | 219.1336,201.1228,173.1285,132.1020,86.0968,60.0450 | HMDB0029042 | 3.60 | peptides | ***↑ |
| 31 | Cysteinylglycine | C5H10N2O3S | [M+H]+ | 2.6035 | 179.0484 | 179.0488,162.0220,116.0167,76.0221,58.9956 | HMDB0000078 | 1.62 | peptides | ***↑ |
| 32 | glu-leu | C11H20N2O5 | [M+H]+ | 8.0803 | 261.1445 | 261.1352,243.1315,198.1124,132.1020,102.0554,86.0969,84.0448 | HMDB0028823 | 4.31 | peptides | ***↑ |
| 33 | gamma-glutamylvaline | C10H18N2O5 | [M+H]+ | 6.7291 | 247.1288 | 247.1289,201.1249,184.0918,118.0864,72.0813 | HMDB0011172 | 2.70 | peptides | ***↑ |
| 34 | creatine | C4H9N3O2 | [M+H]+ | 1.5426 | 132.0768 | 132.0769,90.0554,87.0556,72.0813 | HMDB0000064 | 0.66 | peptides | * ↓ |
| 35 | LPC 16:0/0:0 | C24H50NO7P | [M+H]+ | 18.9051 | 496.3393 | 496.3397,478.3301,184.0733,124.9999,104.1073,86.0969 | HMDB0010382 | 0.42 | phospholipid | ***↓ |
| 36 | LysoPC(0:0/18:1(11Z)) | C26H52NO7P | [M+H]+ | 19.0802 | 522.3552 | 522.3549,184.0733,104.1073,86.0969 | HMDB0010385 | 0.63 | phospholipid | * ↓ |
| 37 | N-acetylputrescine | C6H14N2O | [M+H]+ | 1.7708 | 131.1178 | 131.1180,115.0948,114.0915,72.0813 | HMDB0002064 | 0.79 | polyamine | * ↓ |
| 38 | Hypoxanthine | C5H4N4O | [M+H]+ | 3.0038 | 137.0458 | 137.0458,119.0354,110.0351,94.0403,82.0402 | HMDB0000157 | 0.42 | purines | ***↓ |
| 39 | Xanthine | C5H4N4O2 | [M+H]- | 3.4653 | 151.0256 | 151.0256,133.0149,108.0195 | HMDB0000292 | 0.59 | purines | ** ↓ |
| 40 | Thymine | C5H6N2O2 | [M+H]+ | 4.9463 | 127.0503 | 127.0500,109.0400,84.0448,82.0290,56.0501 | HMDB0000262 | 0.27 | pyrimidines | ***↓ |
| 41 | Folic acid | C19H19N7O6 | [M-H]- | 8.0632 | 440.1325 | 440.1327,422.1220,396.1433,378.1333,310.1042,267.0991,265.0839 | HMDB0000121 | 0.42 | vitamins | ***↓ |

**Reference:**

(1) Cao, J.; Wu, X.; Qin, X.; Li, Z. Uncovering the effect of passage number on HT29 cell line based on the cell metabolomic approach. *J. Proteome Res*. **2021**, *20*, 1582-1590.

(2) Wang, K.-X.; Fang, J.-S.; Qin, X.-M.; Du, G.-H.; Gao, L. Uncovering the anti-metastasis effects and mechanisms of capsaicin against hepatocellular carcinoma cells by metabolomics. *J. Funct. Foods* **2019**, *60*, 103431.
